# Comparison of Serotype Prevalence of Pneumococci Isolated from Middle Ear, Lower Respiratory Tract and Invasive Disease Prior to Vaccination in Iceland

**DOI:** 10.1371/journal.pone.0169210

**Published:** 2017-01-26

**Authors:** Martha Á. Hjálmarsdóttir, Sigríður Júlía Quirk, Gunnsteinn Haraldsson, Helga Erlendsdóttir, Ásgeir Haraldsson, Karl G. Kristinsson

**Affiliations:** 1 Department of Clinical Microbiology, Landspitali University Hospital, Reykjavik, Iceland; 2 Faculty of Medicine, University of Iceland, Reykjavik, Iceland; 3 BioMedical Center of the University of Iceland, Reykjavik, Iceland; 4 Children's Hospital, Landspitali University Hospital, Reykjavik, Iceland; Universidade de Lisboa Faculdade de Medicina, PORTUGAL

## Abstract

**Background:**

Information on pneumococcal serotype distribution before vaccination is a prerequisite for evaluation of vaccine effect. The aim was to investigate the prevalence of pneumococcal serotypes isolated from middle ear (ME), lower respiratory tract (LRT) and from invasive disease (IPD) in Iceland prior to implementation of ten-valent pneumococcal *Haemophilus influenzae* protein D conjugate vaccine (PHiD-CV-10) into the infant vaccination program (April 2011).

**Methods and findings:**

All isolates cultured 2007–2011 from ME, LRT and IPD identified as pneumococci were serotyped and tested for susceptibility at the Clinical Microbiology Department, Landspitali University Hospital that serves approximately 85% of the Icelandic population. Pneumococcal isolates were 1711 and 1616 (94.4%) were available for serotyping and included. Isolates belonging to PHiD-CV10 serotypes (VTs) were 1052 (65.1%). Isolates from ME were 879 (54.4%), with 639 (72.7%) from 0–1 year old patients and 651 of VTs (74%). Isolates from LRT were 564 (34.9%), with 292 (51.8%) from ≥65 years old patients, and 300 (53.2%) of VTs. IPD isolates were 173 (10.7%), although more evenly distributed according to age than isolates from the other sites most were from adults and the youngest age group,101 (58.4%) isolates were of VTs. The most common serotype was 19F, 583 (36.1%). Its prevalence was highest in ME, 400 (45.5%), 172 (30.5%) in LRT and 11 isolates (6.4%), in IPD. Penicillin non-susceptible isolates were 651 (40.3%), mainly belonging to VTs, 611 (93.9%), including 535 (82.2%) of 19F.

**Conclusions:**

Multiresistant isolates of serotype 19F were highly prevalent, especially from ME of young children but also from LRT of adults. Serotype 14 was the most common serotype in IPD. The rate of VTs was high and almost all PNSP were of VTs. There was great difference in vaccine coverage between sampling sites, also reflecting difference in vaccine coverage by age groups.

## Introduction

*Streptococcus pneumoniae* (pneumococcus) causes a range of diseases from mild localized infections like acute otitis media to more severe infections like pneumonia, bacteraemia and meningitis [1]. Pneumococci are also commonly carried in the nasopharynx, especially by young children and spread from there to other parts of the body, or to other individuals [2, 3].

Serotype prevalence is dynamic and depends on many variables, which can be related to pneumococcal properties, host factors, antimicrobial use and vaccination in the community. Furthermore, prevalence may differ according to geographic area and time [4–7]. Serotypes with low invasive potential e.g. 6A, 6B, 14 and 19F most commonly cause non-bacteraemic pneumonia and acute otitis media and are frequently found in the nasopharynx of healthy children [8–10]. Serotypes that are considered to be of high invasive potential, e.g. 1, 4, 7F and 9V, cause invasive disease, but are infrequently found in milder infections and very rarely in carriage [9, 11]. However, serotypes of low invasive potential can be a relatively common cause of invasive disease in high risk individuals, such as young children, the elderly and immunocompromised patients [12].

Pneumococcal disease, both in children and older non-vaccinated individuals, has decreased following childhood vaccinations with protein conjugated vaccines (PCV) and replacement from vaccine to non-vaccine serotypes has been documented [8, 13–15]. At the same time the overall levels of pneumococcal carriage in children remain virtually unchanged, but the types carried are non-vaccine serotypes. [16, 17].

It is important to study serotype prevalence prior to implementation of pneumococcal vaccination programs and to monitor post vaccination changes to evaluate the effect of the vaccine on disease and carriage and changes in the serotype prevalence. The aim was to investigate the prevalence of pneumococcal serotypes isolated from middle ear, lower respiratory tract and from invasive disease in Iceland prior to implementation of ten-valent pneumococcal *Haemophilus influenzae* protein D conjugate vaccine (PHiD-CV-10) into the infant vaccination program.

## Materials and Methods

### Study population

The Department of Microbiology, Landspitali University Hospital, Iceland, serves as the primary microbiology laboratory for the Reykjavik metropolitan area. Furthermore, the laboratory serves as a reference laboratory for the whole country. Inhabitants from other areas of the country often seek health services, both general and specialist services, in the capital and are included in the study. The population of Iceland was 312,872 in 2007 and 318,452 in 2011. In 2011 202,339 inhabitants lived in the metropolitan area. It is estimated that the departments serves 60% of the countryside, thus in that year it served 272,009 (85.4%) inhabitants. Children younger than 7 years old were then 10.1% of the total population and in the metropolitan area 10.7%. (www.statice.is).

The study was approved by the National Bioethics Committee (license no: 12.010.S1).

Vaccination with PHiD-CV10 was initiated in April 2011 thus only part of the children born in that year had received the first vaccinations.

When analysing the data, the patients were divided into the age groups: 0–1, 2–6, 7–17, 18–64 and ≥65 years old.

### Bacterial isolates

The study included all clinical isolates from middle ear (ME), lower respiratory tract (LRT) and invasive diseases (blood, cerebrospinal fluid and joint fluid) (IPD) identified as pneumococci during a five year period, 2007–2011, at the Department of Clinical Microbiology, Landspitali University Hospital. Only one isolate of the same phenotype (defined as identical serotype and antibiogram) from the same patient within 30 days was used. When possible, the first isolate indicating the most severe infection was chosen (IPD, LRT and ME respectively). The isolates were stored in tryptose-glycerol broth at -80°C.

### Antimicrobial susceptibility testing

Antimicrobial susceptibility was performed on all isolates by disk diffusion, against oxacillin, chloramphenicol, erythromycin, trimethoprim-sulfamethoxazole, tetracycline and clindamycin, according to the methods of the Clinical and Laboratory Standards Institute (CLSI) [18, 19] and also interpreted according to the European Committee on Antimicrobial Susceptibility Testing (EUCAST) [20]. Screening for penicillin non-susceptibility was done using 1 μg oxacillin disks. All oxacillin sensitive isolates were defined as sensitive to penicillin, but all resistant isolates were tested for minimum inhibitory concentration to penicillin and ceftriaxone using the E-test (AB-Biodisk) [21]. Isolates with penicillin MIC >0.06mg/L were defined as penicillin non-susceptible.

### Serotyping

All available isolates were serotyped by conventional methods using the Pneumotest kit and/or latex or coagglutination solutions for single antisera (State Serum Institute, Copenhagen) [22, 23] and /or multiplex PCR [10].

A multiplex PCR panel was designed based on previously published methods [24–27]. The serotypes were selected to include the vaccine serotypes that are included in the PHiD-CV10 (VT), the additional serotypes in the 13-valent vaccine and the most common non-vaccine serotypes (NVT) in Iceland, according to our previous studies. The panel consisted of seven multiplex PCR reactions, each with three to four serotype specific primers pairs, in total 27 serogroups/serotypes, and primers for *cpsA*, for the *cps* locus, and *lytA*, for autolysin, that were used for internal positive control. Furthermore, a series of PCR reactions previously described was used to detect serotypes 6A, B,C, D and to separate 6B and its variant 6Bii in all isolates of serogroup 6 [28, 29].

### Statistical analyses

To study association between groups Pearson´s Chi-square and Fisher’s exact test were used. P value <0.05 was considered significant.

## Results

In total, 1711 pneumococcal isolates fulfilled the criteria of the study and of those 1616 (94.4%) were available for serotyping and were included. Distribution according to specimen site and age group did not differ between the available and the 95 (5.6%) non-available (not stored, or non-viable) except for the yield of available isolates from IPD that was higher (99.4%) than for the other sampling sites.

The isolates from ME specimens were 879/1616 (54.4%) and most were from young children. Isolates from 0–1 year old children more often originated from ME than other specimens (p<0.0001). The ME isolates were also more often from 0–1 than 2–6 year old children (p<0.0001). LRT isolates were 564/1616 (34.9%) and most were from adults. Isolates from ≥65 year old patients were more commonly from LRT than the other sampling sites (p<0.0001). The isolates from IPD specimens were 173/1616 (10.7%), although more evenly distributed according to age than isolates from the other sites most were from adults and the youngest age group (Table 1).

**Table 1:** Distribution of all isolates and PNSP isolates according to sampling site and age of the patient and their proportions (%) within age group and sampling site.

Age	ME		LRT		IPD		Total	
group	All n (%)	PNSP n (%)	All n (%)	PNSP n (%)	All n (%)	PNSP n (%)	All n (%)	PNSP n (%)
0–1	639 (72.7)	353 (55.2)	8 (1.4)	3 (37.5)	26 (15.0)	5 (19.2)	**673 (42.7)**	**361 (53.6)**
2–6	209(23.8)	68 (32.5)	25 (4.4)	11 (44.0)	14 (8.1)	0	**248 (23.8)**	**79 (31.9)**
7–17	14 (1.6)	1 (7.1)	13 (2.3)	6 (46.2)	4 (2.3)	0	**31 (1.9)**	**7 (22.6)**
18–64	14 (1.6)	5 (35.7)	226 (40.1)	76 (33.6)	69 (39.9)	5 (7.2)	**309 (19.1)**	**86 (27.8)**
≥65	3 (0.3)	1 (33.3)	292 (51.8)	109 (37.3)	60 (34.7)	8 (13.3)	**355 (22.1)**	**118 (33.2)**
**Total**	**879 (54.4)**	**428 (48.7)**	**564 (34.9)**	**205 (36.3)**	**173 (10.7)**	**18 (10.4)**	**1616**	**651 (40.3)**

Temporal changes in number of pneumococcal isolates were noted, the highest number of isolates was in 2007, 357 (22.1%) and gradually declined to 283 in 2011 (17.5%).

### Prevalence of serotypes

The isolates were of 52 serotypes. The most common was serotype 19F with 583/1616 (36.1%) isolates, followed by 23F with 157/1616 (9.7%) (Table 2). Of all isolates, 1052/1616 (65.1%) belonged to VT-10 and were significantly more common than non-vaccine serotypes (NVT-10) (P<0.0001).

**Table 2:** Ranking of the eight most common serotypes according to infection site and their proportions (%) and of VTs and NVTs according to sampling site.

ME		LRT		IPD		Total	
Type	n (%)	Type	n (%)	Type	n (%)	Type	n (%)
19F	400 (45.5)	19F	172 (30.5)	14	28 (16.2)	19F	583 (36.1)
23F	111 (12.6)	3	49 (8.7)	19A	17 (9.8)	23F	157 (9.7)
6A	73 (8.3)	6A	42 (7.4)	4	14 (8.1)	6A	122 (7.5)
14	61 (6.9)	23F	40 (7.1)	9V	13 (7.5)	14	117 (7.2)
19A	61 (6.9)	6B	32 (5.7)	7F	12 (6.9)	6B	101 (6.3)
6B	60 (6.8)	14	28 (5.0)	19F	11 (6.4)	19A	99 (6.1)
3	28 (3.2)	NT	23 (4.1)	3	9 (5.2)	3	86 (5.3)
9V	14 (1.6)	19A	21 (3.7)	6B	9 (5.2)	9V	47 (2.9)
Other	71 (8.1)	Other	157 (27.9)	Other	60 (34.7)	Other	304 (18.8)
**Total**	**879 (54.4)**	**Total**	**564 (34.9)**	**Total**	**173 (10.7)**	**Total**	**1616**
VT-10	651 (74.1)	VT	300 (53.2)	VT	101 (58.4)	VT	1052 (65.1)
NVT-10	228 (25.9)	NVT	264 (46.8)	NVT	72 (41.6)	NVT	564 (34.9)
VT-13	813 (92.5)	VT-13	412 (73.0)	VT-13	134 (77.5)	VT-13	1359 (84.1)
NVT-13	66 (7.5)	NVT-13	152 (27.0)	NVT-13	39 (22.5)	NVT-13	257 (34.9)

n = the number of isolates.

NT = unencapsulated isolates, negative in PCR reactions using *cpsA* and serotype specific primers, positive in *lyt*A reactions. Other possibilities might be serotypes 25F/A, or 38, known for changes in the *cps*A gene causing negative reactions.

The ME isolates were of 34 serotypes and the most common was serotype 19F with 400/879 (45.5%) isolates and significantly more common than in LRT and IPD (P<0.0001). The prevalence of the 8 most common serotypes ranged from 45.5–1.6% (Table 2). The proportion of isolates belonging to VTs was highest in ME, 651/879 (74.1%). The LRT isolates were of 45 serotypes and the most common was 19F with 172/564 (30.5%) isolates. The prevalence of the 8 most common serotypes ranged from 30.5–3.7%. The proportion of isolates belonging to VT-10 was lowest in LRT, 300/564 (53.2%). The IPD isolates were of 29 serotypes and the most common serotype was serotype 14 with 28/173 (16.2%) isolates. The prevalence of the 8 most common serotypes ranged from 16.2–4.6% (Table 2). The isolates belonging to VTs were 101/173 (58.4%) (Table 2). Overall, the most common NVTs were 11A, 41 isolates (2.5%) and 33D, 18 (1.1%) isolates.

### Antimicrobial susceptibility

Penicillin non-susceptible pneumococcal isolates (PNSP) were 651/1616 (40.3%) (Table 1). The number of PNSP isolates remained stable during the study period, 128–138 isolates each year, while the number of all isolates gradually decreased from 357–283. Accordingly, the PNSP proportions gradually increased from 36.7% in 2007 to 44.9% in 2011, resulting in significant difference between the first and last year (p = 0.035). PNSP isolates of VT-10 were 611/651 (93.9%). Similar temporal changes in the rates of VTs of PNSP were seen but with more fluctuations between years.

PNSP isolates were 428/879 (48.7%) of ME, 205/564 (36.3%) of LRT and 18/173 (10.4%) of IPD isolates. PNSP of VTs were significantly more common in ME than in LRT (p = 0.0001) and also more common than in IPD (p = 0.0025). The VTs were significantly more common than the NVTs among PNSP in all instances (Table 3).

**Table 3:** Ranking of the 4 most common PNSP serotypes according to sampling site.

ME		LRT		IPD		Total	
Type	n (%)	Type	n (%)	Type	n (%)	Type	n (%)
19F	378 (88.3)	19F	150 (73.2)	19F	7 (38.9)	19F	535 (82.2)
14	12 (2.8)	NT	13 (6.3)	9V	2 (11.1)	6B	32 (4.9)
6B	19 (4.4)	6B	12 (5.9)	14	2 (11.1)	14	20 (3.1)
19A	7 (1.6)	9V	10 (4.9)	23F	2 (11.1)	9V	14 (2.2)
Other	12 (2.8)	Other	20 (9.8)	Other	5 (27.8)	Other	50 (7.7)
**Total**	**428 (65.7)**	**Total**	**205 (31.5)**	**Total**	**18 (2.8)**	**Total**	**651**
VT	416 (97.2)	VT	181 (88.3)	VT	14 (77.8)	VT	611 (93.9)
NVT	10 (2.8)	NVT	24 (11.7)	NVT	4 (22.2)	NVT	40 (6.1)

n = the number of isolates

The PNSP were of 18 serotypes with serotype 19F being the most common, 535/651 (82.2%) isolates. Serotype 19F was the most common PNSP serotype in all specimen groups (p<0.0001), most commonly found in ME with 378/428 (88.3%), in LRT 150/205 (73.2%) and in IPD 7/18 (38.9%) of PNSP isolates (Table 3). The majority of the serotype 19F isolates, or 501/535 (93.6%) isolates had identical antibiograms and were multi-resistant. Their penicillin MIC was close to the breakpoint between intermediate and resistant (median MIC 1.0 μg/mL), thus either defined as intermediate or resistant, resistant to erythromycin, tetracycline and trimethoprim-sulfamethoxazole and sensitive to chloramphenicol and clindamycin as previously described [7]. The remaining 34 (6.4%) isolates of PNSP serotype 19F had diverse antibiograms. Isolates of 6B sharing the antibiograms of the previously dominating clone in the country, of the variant 6Bii of serotype 6B [30] were 24 (1.5%).

## Discussion

Serotype 19F was the dominant serotype, with more than a third of all isolates and more than four of every five PNSP isolates belonging to 19F. It had a predilection for middle ear where most of the samples originated from young children. The serotype was also the dominant PNSP type and almost all the isolates had identical antibiogram. The vast majority of isolates of PNSP belonged to VTs.

The ME isolates were from patients with perforated tympanic membranes or with tympanic tubes, therefore mainly representing acute otitis media which is most common in children. Accordingly, it must be taken into account that when we compare isolates from the different sites, it not only highlights differences between different diseases but also different age groups. Almost half of the isolates belonged to serotype 19F that was the most common and two thirds of all isolates of serotype 19F originated from ME. The vast majority of the 19F isolates were multi-resistant and shared identical antibiograms with the previously described single and double locus variant of the Taiwan^19F^-14 clone [7]. Other isolates belonged to serotypes commonly found in otitis media and carriage and isolates with high invasive potential were rarely seen [11, 12]. Besides the exceptionally high rate of 19F our findings were in concordance with other studies [9, 31–36]. The reason for this high rate might be related to a relatively high antimicrobial usage in the country, making the environment favourable for this multiresistant piliated clone [37]. The high prevalence of VTs and PNSP in ME compared to LRT and IPD can mostly be explained with the exceptionally high rate of 19F in ME samples.

The LRT isolates were mainly isolated from sputum samples and bronchoalveolar lavage fluids, specimens that are rarely obtained from children. Therefore, these isolates are mainly from adults, especially the elderly. The age distribution reflects high risk groups for pneumococcal pneumonia except for young children. Close to a third of the isolates belonged to serotype 19F that was the most common, but isolates of high invasive potential were rarely seen. The rate of isolates of VTs was lower in LRT than in the other specimen groups. Most likely this reflects the high diversity of the serotypes found in LRT isolates, lower rate of 19F than in ME and compared to IPD low rate of isolates of high invasive serotypes that are VTs. More than a third of the isolates were PNSP, most of serotype 19F.

About half the IPD isolates were from the youngest and the oldest age groups. The two most common serotypes were 14 and 19A. This is of interest as these serotypes are generally considered to be of a low or moderate invasive potential [12, 38]. The reason for this remains unclear. It may be related to clonal properties as different clones of serotype 14 have been reported to be more prevalent in IPD than carriage and vice versa [12]. Following vaccination with the 7-valent conjugative vaccine serotype 19A was commonly reported in IPD [39, 40]. It is possible that the effect of vaccination in neighbouring countries may have had an indirect effect on serotype distribution in IPD in this country. Another possible explanation for the relatively common IPD by these less invasive serotypes is the patient’s age. Young children and older individuals have less competent immune system. In addition, older people may more often be immunocompromised due to co-morbidity or medical therapy. This weak immune system may certainly render the individuals more prone to infections, even with less invasive serotypes [41]. The next three serotypes in the rank, serotypes 4, 7F and 9V are considered highly invasive [4, 11, 42, 43]. The age distribution of the patients diagnosed with the highly invasive serotypes tended to be more evenly distributed which is in concordance with our explanations and other studies [5, 12]. Serotypes that are commonly carried by children, or known for causing milder disease, were a relatively common cause for IPD [8–10].

The PNSP rates were high and mostly seen in serotypes that are frequent colonizers and more often in ME than in other infections. This is in concordance with other studies showing that antimicrobial resistance is more likely to be found in milder infections than in invasive disease, with the exception of individuals at high risk [44]. The outpatient usage of antimicrobials in Iceland is relatively high and brings selective pressure for resistant clones that along with the properties of successful clones can influence the rates of serotypes [7, 37]. However, the vast majority of PNSP were of VTs and vaccination is therefore likely to reduce the PNSP rates substantially.

In summary, the rates of serotype 19F were very high, especially in isolates from ME that were mostly from young children. 19F was also the dominant serotype in isolates from LRT that were mainly from adults and the elderly. The vast majority of the 19F isolates were multiresistant, with identical antibiograms. The most common serotype in IPD was serotype 14. The rate of vaccine serotypes was highest in isolates from middle ear and lowest in isolates from lower respiratory tract, where serotype diversity was the most. Penicillin non-susceptible isolates were almost solely of vaccine serotypes. There was great difference in vaccine coverage between sampling sites, also reflecting difference in vaccine coverage by age groups. These results provide important information on serotype distribution and antibiotic resistance prior to immunization and will serve as a basis for evaluation of the effect of infant pneumococcal vaccination.

## Supporting Information

S1 Table This is the full supporting information that includes impersonalized relevant information of specimen aquisition and results. (XLSX)

## References

[1] Austrian R. Pneumococcus: the first one hundred years. Rev Infect Dis. 1981;3(2):183–9. 10.1093/clinids/3.2.183 7020040

[2] Simell B, Auranen K, Käyhty H, Goldblatt D, Dagan R, O'Brien KL. The fundamental link between pneumococcal carriage and disease. Expert Rev Vaccines. 2012;11(7):841–55. 10.1586/erv.12.53 22913260

[3] Leiberman A, Dagan R, Leibovitz E, Yagupsky P, Fliss DM. The bacteriology of the nasopharynx in childhood. Int J Pediatr Otorhinolaryngol. 1999;49 Suppl 1:S151–3. 10577795 10.1016/s0165-5876(99)00151-2

[4] Alanee SRJ, McGee L, Jackson D, Chiou CC, Feldman C, Morris AJ, et al Association of serotypes of *Streptococcus pneumoniae* with disease severity and outcome in adults: An international study. Clin Infect Dis. 2007;45(1):46–51. 10.1086/518538 17554699

[5] Cartwright K. Pneumococcal disease in western Europe: burden of disease, antibiotic resistance and management. Eur J Pediatr. 2002;161(4):188–95. 12014384 10.1007/s00431-001-0907-3

[6] Dagan R. New insights on pneumococcal disease: What we have learned over the past decade. Vaccine. 2009;27(SUPPL. 3):C3–C5. 19552985 10.1016/j.vaccine.2009.06.002

[7] Hjalmarsdottir MA, Kristinsson KG. Epidemiology of penicillin-non-susceptible pneumococci in Iceland, 1995–2010. J Antimicrob Chemother. 2014;69(4):940–6. 10.1093/jac/dkt470 24311742

[8] Horácio AN, Lopes JP, Ramirez M, Melo-Cristino J. Non-invasive pneumococcal pneumonia in Portugal—serotype distribution and antimicrobial resistance. PLoS One. 2014;9(7). 10.1371/journal.pone.0103092 PMC4116175 25075961

[9] Alonso M, Marimon JM, Ercibengoa M, Pérez-Yarza EG, Pérez-Trallero E. Dynamics of *Streptococcus pneumoniae* serotypes causing acute otitis media isolated from children with spontaneous middle-ear drainage over a 12-year period (1999–2010) in a region of northern Spain. PLoS ONE. 2013;8(1). 10.1371/journal.pone.0054333 PMC3551958 23349853

[10] Hjalmarsdottir MA, Gumundsdottir PF, Erlendsdottir H, Kristinsson KG, Haraldsson G. Cocolonization of pneumococcal serotypes in healthy children attending day care centers: Molecular versus conventional methods. Pediatr Infect Dis J. 2016;35(5):477–80. 10.1097/INF.0000000000001059 26808723

[11] Brueggemann AB, Peto TE, Crook DW, Butler JC, Kristinsson KG, Spratt BG. Temporal and geographic stability of the serogroup-specific invasive disease potential of *Streptococcus pneumoniae* in children. J Infect Dis. 2004;190(7):1203–11. 10.1086/423820 15346329

[12] Sandgren A, Sjöström K, Olsson-Liljequist B, Christensson B, Samuelsson A, Kronvall G, et al Effect of clonal and serotype-specific properties on the invasive capacity of *Streptococcus pneumoniae*. J Infect Dis. 2004;189(5):785–96. 10.1086/381686 14976594

[13] Imöhl M, René Reinert R, van der Linden M. Serotype-specific penicillin resistance of *Streptococcus pneumoniae* in Germany from 1992 to 2008. Int J Med Bicrob. 2010;300(5):324–30. 10.1016/j.ijmm.2009.11.004 20071233

[14] Regev-Yochay G, Paran Y, Bishara J, Oren I, Chowers M, Tziba Y, et al Early impact of PCV7/PCV13 sequential introduction to the national pediatric immunization plan, on adult invasive pneumococcal disease: A nationwide surveillance study. Vaccine. 2015;33(9):1135–42. 10.1016/j.vaccine.2015.01.030 25613717

[15] Beall BW, Gertz RE, Hulkower RL, Whitney CG, Moore MR, Brueggemann AB. Shifting genetic structure of invasive serotype 19A pneumococci in the United States. J Infect Dis. 2011;203(10):1360–8. 10.1093/infdis/jir052 21398395 PMC3080895

[16] Rodrigues F, Nunes S, Sa-Leao R, Goncalves G, Lemos L, de Lencastre H. *Streptococcus pneumoniae* nasopharyngeal carriage in children attending day-care centers in the central region of Portugal, in the era of 7-valent pneumococcal conjugate vaccine. Microb Drug Resist. 2009;15(4):269–77. 10.1089/mdr.2009.0043 19857133

[17] Ricketson LJ, Wood ML, Vanderkooi OG, MacDonald JC, Martin IE, Demczuk WH, et al Trends in asymptomatic nasopharyngeal colonization with *Streptococcus pneumoniae* after introduction of the 13-valent pneumococcal conjugate vaccine in Calgary, Canada. Pediatr Infect Dis J. 2014;33(7):724–30. 10.1097/INF.0000000000000267 24463806

[18] CLSI. Performance Standard for Antimicrobial Susceptibility Testing; Seventh Informational Supplement. Wayne, PA: Clinical and Laboratory Standards Institute; 2007.

[19] CLSI. Performance Standard for Antimicrobial Disk Susceptibility Tests; Approved Standard—Tenth Edition. Wayne, PA: Clinical and Laboratory Standards Institute; 2009.

[20] Breakpoint tables for interpretation of MICs and zone diameters [Internet]. The European Commitee on Antimicrobial Susceptiblility Testing. 2012 [cited 2012-06-01]. Available from: http://www.eucast.org/fileadmin/src/media/PDFs/EUCAST_files/Breakpoint_tables/Breakpoint_table_v_2.0_120221.xls.

[21] AB-Biodisk. Susceptibility testing of pneumococci, vol. E-test technical guide 5C. Solna. Sweden: AB-Biodisk; 1998.

[22] Sørensen UB. Typing of pneumococci by using 12 pooled antisera. J Clin Microbiol. 1993;31(8):2097–100. 8370735 10.1128/jcm.31.8.2097-2100.1993 PMC265703

[23] Slotved HC, Kaltoft M, Skovsted IC, Kerrn MB, Espersen F. Simple, rapid latex agglutination test for serotyping of pneumococci (Pneumotest-Latex). J Clin Microbiol. 2004;42(6):2518–22. 10.1128/JCM.42.6.2518-2522.2004 15184429 PMC427861

[24] Pai R, Gertz RE, Beall B. Sequential multiplex PCR approach for determining capsular serotypes of *Streptococcus pneumoniae* isolates. J Clin Microbiol. 2006;44(1):124–31. 10.1128/JCM.44.1.124-131.2006 16390959 PMC1351965

[25] Sourav S, Patricia A, Sharma S, Kanungo R, Jayachandran S, Prashanth K. Detection of pneumolysin and autolysin genes among antibiotic resistant *Streptococcus pneumoniae* in invasive infections. Indian J Med Microbiol. 2010;28(1):34–9. 10.4103/0255-0857.58726 20061761

[26] Dobay O, Juhasz E, Ungvari A, Jeney C, Amyes SG, Nagy K. Taguchi optimisation of a multiplex pneumococcal serotyping PCR and description of 11 novel serotyping primers. Acta Microbiol Immunol Hung. 2009;56(4):327–38. 10.1556/AMicr.56.2009.4.3 20038485

[27] Zhou F, Kong F, Tong Z, Gilbert GL. Identification of less-common *Streptococcus pneumoniae* serotypes by a multiplex PCR-based reverse line blot hybridization assay. J Clin Microbiol. 2007;45(10):3411–5. 10.1128/JCM.01076-07 17687009 PMC2045372

[28] Jin P, Xiao M, Kong F, Oftadeh S, Zhou F, Liu C, et al Simple, accurate, serotype-specific PCR assay to differentiate Streptococcus pneumoniae serotypes 6A, 6B, and 6C. J Clin Microbiol. 2009;47(8):2470–4. 10.1128/JCM.00484-09 19535528 PMC2725661

[29] Kawaguchiya M, Urushibara N, Kobayashi N. High prevalence of genotype 6E (putative serotype 6E) among noninvasive/colonization isolates of *Streptococcus pneumoniae* in northern Japan. Microb Drug Resist. 2015;21(2):209–14. 10.1089/mdr.2014.0181 25361198

[30] van Tonder AJ, Bray JE, Roalfe L, White R, Zancolli M, Quirk SJ, et al Genomics Reveals the Worldwide Distribution of Multidrug-Resistant Serotype 6E pneumococci. J Clin Microbiol. 2015;53(7):2271–85. 10.1128/JCM.00744-15 25972423 PMC4473186

[31] Mavroidi A, Paraskakis I, Pangalis A, Kirikou E, Charisiadou A, Athanasiou T, et al Spread of the *Streptococcus pneumoniae* Taiwan^19F^-14 clone among children in Greece. Clin Microbiol Infect. 2007;13(12):1213–6. 10.1111/j.1469-0691.2007.01837.x 17944968

[32] Hanage WP, Auranen K, Syrjänen R, Herva E, Mäkelä PH, Kilpi T, et al Ability of pneumococcal serotypes and clones to cause acute otitis media: Implications for the prevention of otitis media by conjugate vaccines. Infect Immun. 2004;72(1):76–81. 10.1128/IAI.72.1.76-81.2004 14688083 PMC343969

[33] McEllistrem MC, Adams JM, Patel K, Mendelsohn AB, Kaplan SL, Bradley JS, et al Acute otitis media due to penicillin-nonsusceptible *Streptococcus pneumoniae* before and after the introduction of the pneumococcal conjugate vaccine. Clin Infect Dis. 2005;40(12):1738–44. 10.1086/429908 15909260

[34] Reijtman V, Fossati S, Hernandez C, Sommerfleck P, Bernaldez P, Litterio M, et al Serotype distribution of pneumococci isolated from pediatric patients with acute otitis media and invasive infections, and potential coverage of pneumococcal conjugated vaccines. Rev Argent Microbiol. 2013;45(1):27–33. 23560785

[35] Mayanskiy N, Alyabieva N, Ponomarenko O, Pakhomov A, Kulichenko T, Ivanenko A, et al Bacterial etiology of acute otitis media and characterization of pneumococcal serotypes and genotypes among children in Moscow, Russia. Pediatr Infect Dis J. 2015;34(3):255–60. 10.1097/INF.0000000000000554 25232779

[36] Pan F, Han L, Huang W, Tang J, Xiao S, Wang C, et al Serotype Distribution, Antimicrobial Susceptibility, and Molecular Epidemiology of *Streptococcus pneumoniae* Isolated from Children in Shanghai, China. PLoS One. 2015;10(11). 10.1371/journal.pone.0142892 PMC4646667 26571373

[37] Hjalmarsdottir MA, Petursdottir B, Erlendsdottir H, Haraldsson G, Kristinsson KG. Prevalence of pilus genes in pneumococci isolated from healthy preschool children in Iceland: association with vaccine serotypes and antibiotic resistance. J Antimicrob Chemother. 2015;70(8):2203–8. 10.1093/jac/dkv096 25888572

[38] Brueggemann AB, Griffiths DT, Meats E, Peto T, Crook DW, Spratt BG. Clonal relationships between invasive and carriage *Streptococcus pneumoniae* and serotype- and clone-specific differences in invasive disease potential. J Infect Dis. 2003;187(9):1424–32. 10.1086/374624 12717624

[39] Choi EH, Kim SH, Eun BW, Kim SJ, Kim NH, Lee J, et al *Streptococcus pneumoniae* serotype 19A in children, South Korea. Emerg Infect Dis. 2008;14(2):275–81. 10.3201/eid1402.070807 18258121 PMC2600206

[40] Kaplan SL, Barson WJ, Lin PL, Stovall SH, Bradley JS, Tan TQ, et al Serotype 19A Is the most common serotype causing invasive pneumococcal infections in children. Pediatrics. 2010;125(3):429–36. 10.1542/peds.2008-1702 20176669

[41] Henriques-Normark B, Tuomanen EI. The pneumococcus: epidemiology, microbiology, and pathogenesis. Cold Spring Harb Perspect Med. 2013;3(7). 10.1101/cshperspect.a010215 PMC3685878 23818515

[42] Hanage WP, Kaijalainen TH, Syrjanen RK, Auranen K, Leinonen M, Makela PH, et al Invasiveness of serotypes and clones of *Streptococcus pneumoniae* among children in Finland. Infect Immun. 2005;73(1):431–5. 10.1128/IAI.73.1.431-435.2005 15618181 PMC538975

[43] Henriques-Normark B, Blomberg C, Dagerhamn J, Battig P, Normark S. The rise and fall of bacterial clones: *Streptococcus pneumoniae*. Nat Rev Microbiol. 2008;6(11):827–37. 10.1038/nrmicro2011 18923410

[44] Sjöström K, Spindler C, Ortqvist A, Kalin M, Sandgren A, Kuhlmann-Berenzon S, et al Clonal and capsular types decide whether pneumococci will act as a primary or opportunistic pathogen. Clin Infect Dis. 2006;42(4):451–9. 10.1086/499242 16421787

